# Virtual Reality Technology Use in Cigarette Craving and Smoking Interventions (I “Virtually” Quit): Systematic Review

**DOI:** 10.2196/24307

**Published:** 2021-09-17

**Authors:** Merel Keijsers, Maria Cecilia Vega-Corredor, Melanie Tomintz, Simon Hoermann

**Affiliations:** 1 School of Product Design College of Engineering University of Canterbury Christchurch New Zealand; 2 HIT Lab NZ College of Engineering University of Canterbury Christchurch New Zealand; 3 John Cabot University Rome Italy; 4 Geospatial Research Institute University of Canterbury Christchurch New Zealand

**Keywords:** virtual reality technology, nicotine dependency, nicotine addiction, smoking addiction, smoking intervention, smoking therapy, Electronic Nicotine Delivery Systems

## Abstract

**Background:**

Over the last 2 decades, virtual reality technologies (VRTs) have been proposed as a way to enhance and improve smoking cessation therapy.

**Objective:**

This systematic review aims to evaluate and summarize the current knowledge on the application of VRT in various smoking cessation therapies, as well as to explore potential directions for future research and intervention development.

**Methods:**

A literature review of smoking interventions using VRT was conducted.

**Results:**

Not all intervention studies included an alternative therapy or a placebo condition against which the effectiveness of the intervention could be benchmarked, or a follow-up measure to ensure that the effects were lasting. Virtual reality (VR) cue exposure therapy was the most extensively studied intervention, but its effect on long-term smoking behavior was inconsistent. Behavioral therapies such as a VR approach-avoidance task or gamified interventions were less common but reported positive results. Notably, only 1 study combined Electronic Nicotine Delivery Devices with VRT.

**Conclusions:**

The inclusion of a behavioral component, as is done in the VR approach-avoidance task and gamified interventions, may be an interesting avenue for future research on smoking interventions. As Electronic Nicotine Delivery Devices are still the subject of much controversy, their potential to support smoking cessation remains unclear. For future research, behavioral or multicomponent interventions are promising avenues of exploration. Future studies should improve their validity by comparing their intervention group with at least 1 alternative or placebo control group, as well as incorporating follow-up measures.

## Introduction

### Background

Smoking addiction is a worldwide [1] and costly [2] problem. Tobacco smoking has been linked to cardiovascular disease [3], various types of cancer [4-6] and respiratory problems [7], among other diseases, reducing the consumers’ quality of life [8], and lowering life expectancy by up to 4 years [9]. Approximately 30% of all cancer deaths in high-income countries are caused by cigarette smoking [10].

The Diagnostic and Statistical Manual of Mental Disorders lists nicotine dependency (rephrased as tobacco use disorder in a more recent edition) as a psychological disorder [11]; 55% of US smokers try to quit every year [12], but it has been estimated that between 60% and 90% of them relapse within the first year [12-14]. Smoking addiction has a strong psychological component [15,16] in addition to physiological dependence. Physiological craving may only take a few weeks to wane [17], but the habit and psychological link between smoking, socializing, relaxing, and rewarding are much harder to abandon [16]. Thus, different therapies and interventions have been developed to overcome this link.

### Interventions and Therapies

Therapies and interventions are both care strategies, but their main difference is the scope. Interventions usually aim to motivate someone to commit to a specific action, such as a teenager saying *no* when their peers offer a cigarette or a smoker entering treatment. Therapies, on the other hand, address more comprehensive goals, such as maintaining abstinence and altering habits or attitudes. In practice, there is often no clear distinction between the two and they can be thought of as lying on a continuum rather than reflecting 2 dichotomous categories [18].

Cue exposure therapy (CET) uses a classical conditioning approach to unlearn a response (craving) to stimuli (smoking-related items and situations). During the therapy sessions, craving is elicited by exposing participants to smoking-related cues such as cigarette packages and images of situations in which the participant usually smoked. In daily life, participants would relieve cravings through smoking, which reinforces the craving response. During CET, typically no nicotine reward is provided [19] (however, see the study by Kotlyar et al [20] and De La Garza et al [21] that combines CET with nicotine replacement therapy [NRT]). Once the craving has receded, the participant is exposed to smoking cues again, with the aim of repeating this procedure until the association between smoking cues and reward is weakened and eventually extinguished [19,22].

The approach-avoidance task (AAT) adopts an operant conditioning approach. This task can be used to measure and influence subconscious bias [23,24]. During the AAT, participants have to either pull or push away a lever in response to visual cues. People tend to be quicker to pull when presented with cues that elicit a positive (approach) bias than when cues have a neutral or negative association, whereas they are quicker to push away (avoid) negative cues than positive or neutral ones [23]. Thus, AAT can be used to measure existing subconscious biases, as smokers will be quicker to pull when presented with smoking-related cues than nonsmokers. In addition, AAT can be used as an instrument to break or reverse subconscious biases by instructing participants to respond to smoking cues with avoidance behaviors (eg, pushing away) over a number of trials or sessions until smoking cues are automatically associated with avoidance and negative affect [24,25].

Cognitive behavioral therapy (CBT)–based interventions often take a multicomponent approach: teaching smokers to recognize the thought patterns they engage in before smoking and to identify triggering factors in the environment. Smokers are then trained in alternative strategies to cope with craving and temptation [26].

NRT aims to reduce cravings and by extension smoking behavior by replacing the source of nicotine [27]. It is an accessible standalone intervention for smokers who do not have the resources or motivation to commit to a more substantial intervention but will not target the psychological component of the smoking habit [27]. To this effect, NRT is often combined with CBT [28,29].

One particular form of NRT includes the use of Electronic Nicotine Delivery Systems (ENDS), more commonly known as electronic cigarettes, e-cigarettes, or vapes. Invented at the start of the 2000s, these devices heat a solution usually containing nicotine and flavoring agents and deliver the vapor (aerosol) to the user to be inhaled [30], although the amount of nicotine varies and nonnicotine liquids are available as well [31,32]. ENDS were originally introduced as a device to help reduce the number of cigarettes smoked or even quit (conventional) smoking altogether [30,33,34]. However, there has been an ongoing debate in the scientific and medical community on the health risks associated with ENDS [35,36] as well as the potential of ENDS to form an introduction to smoking for previous nonsmokers (the gateway hypothesis; [37]). Recent meta-analyses suggest that although far from harmless, ENDS may still pose a lower health risk than smoking cigarettes [37,38] and that they may be more effective than NRT for smoking cessation [39]. The question of whether this makes ENDS an eligible form of NRT has yet to be resolved [40,41].

Virtual reality technologies (VRTs) have been recognized as potentially helpful in increasing the effects of these and other interventions. These technologies provide an immersive interface that can be used to enhance (augmented reality [AR]) or even replace (virtual reality [VR]) reality with computer-generated simulations. AR is commonly used on a screen that combines the display of the real world with some added virtual features; a well-known example is the game Pokémon Go (Niantic Inc), which displayed the camera view on screen but added virtual Pokémon creatures to the scene, which the user could interact with. In contrast, in VR apps, users often wear a VR headset such as a stereoscopic head-mounted display that projects video images in 3D. Although the focus in VR and AR generally lies in visual simulation, the experience can be enhanced through haptic, olfactory, and audio feedback.

There are multiple potential benefits of using the VRT. First, training smokers to respond to a potentially tempting situation will be more effective if the therapy can mimic those situations more closely [42,43], and VR has been shown to create a stronger feeling of being immersed than 2D images [44-46]. Second, VR offers a safe environment for coping skills [47]. Finally, it can be easily tailored to the specific needs of individual smokers [22,42]. Therefore, over the last 15 years, the potential of VR for smoking cessation therapy has been extensively studied. Although AR has not yet been widely embraced, it was still included in the current search strategy.

### This Research

In this paper, we systematically review and summarize the findings from the literature on the adoption of VRT in smoking cessation therapy.

This paper is centered around three main research questions:

- Has VRT been used satisfactorily to elicit smoking cravings?
- What VRT interventions exist and how do they compare with regular interventions in terms of smoking cessation outcomes?
- What are the potential future directions for VRT in smoking cessation therapy?

The review will focus on the adult population (people aged ≥18 years) of smokers with no comorbidities. Randomized controlled trials, controlled trials, single group pre- and posttest studies, and case studies were all included, as well as protocols (to give an indication of future directions of research), meta-analyses (as those can detect effects with greater power than individual studies), and reviews (for the reference list search and to refer to as an overview for the interested reader). Intervention studies were included if they incorporated VRT in their intervention and measured either smoking cue reactivity or intervention effectiveness. With regard to the data extracted, the comparators were treatment, placebo, pre- and postcomparison, and waiting list. The outcomes were craving or smoking urge, nicotine dependence, number of cigarettes smoked, abstinence rates, and quit rates.

Some systematic review papers have been published [48-52], but their scopes only partially overlapped with that of this one; for example, some systematic reviews included nonadult subjects [50], focused exclusively on cue reactivity or CET [48-50,52], limited themselves to head-mounted display VR [51], studied a variety of addictions [48,50,51], or were simply published over 5 years ago, thus missing more recent publications [49,50]. This paper includes 15 papers that were not covered by the previous reviews and thus expands on previous findings by presenting a wider and updated overview of the potential of VRT in smoking cessation.

## Methods

### Search Criteria

The PRISMA (Preferred Reporting Items for Systematic Reviews and Meta-Analyses) guidelines for review [53] were followed for the current review. The database search strategy was developed under the supervision of a trained subject librarian. After deliberation with the librarian, 5 databases (MEDLINE, Embase, Scopus, Cochrane, and EbscoHost) were identified to be searched using the following search terms: (*Virtual reality*, OR *Augmented reality*, OR *Mixed Reality*, OR *Augmented Virtuality*) AND (*smoking* OR *vaping* OR *tobacco* OR *cigarette* OR *nicotine* OR *vape* OR *e-cigarette*) AND (*craving* OR *crave* OR *withdrawal* OR *cue exposure* OR *cue reactivity* OR *urge* OR *cessation* OR *desire*). The reference lists of selected papers were also scanned for other relevant papers.

### Literature Selection

**Figure 1:**
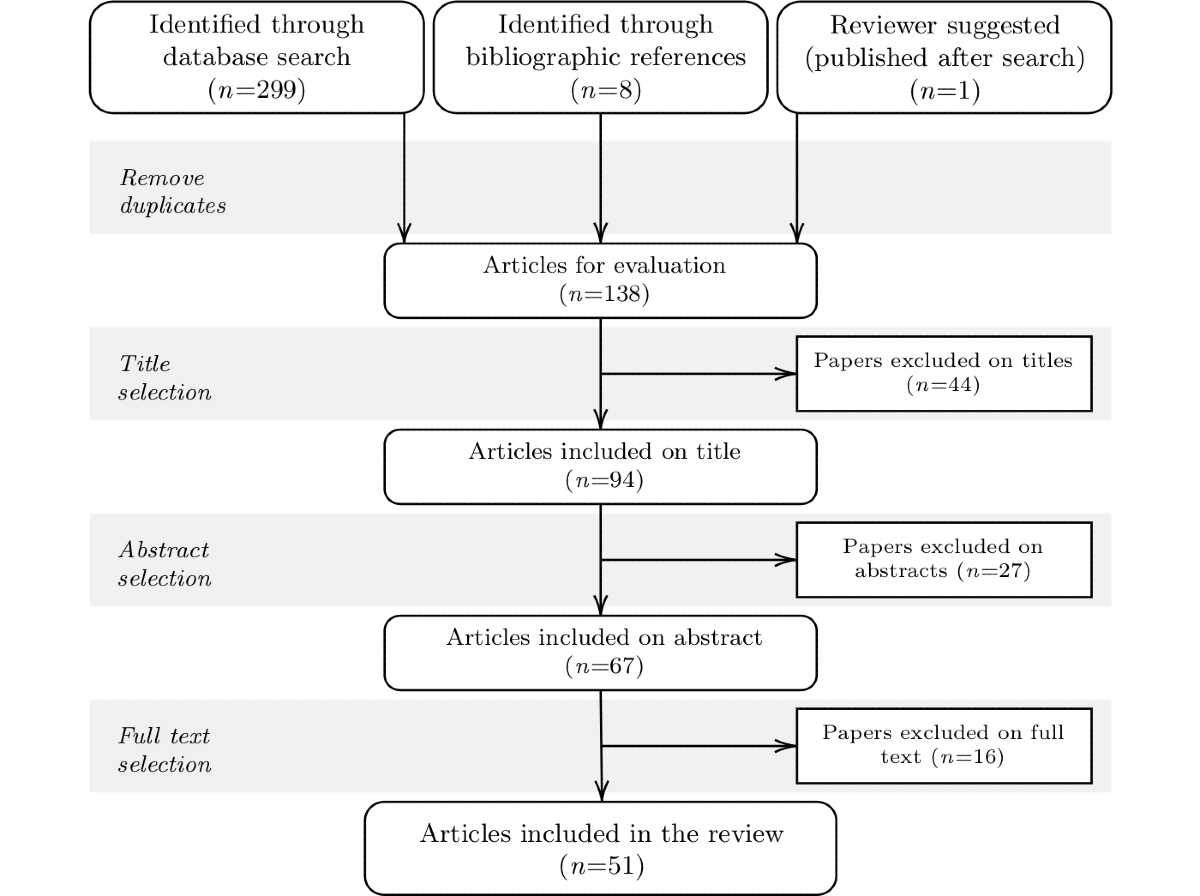
Overview of the screening and paper selection process.

The search was completed in July 2020, resulting in a total of 299 papers: MEDLINE (n=51), Embase (n=51), Scopus (n=88), Cochrane (n=52), and EbscoHost (n=57). After removing duplicates, 137 papers remained. Three rounds of selection were completed: exclusion based on title, exclusion based on the abstract, and exclusion based on the entirety of the paper. Inclusion and exclusion criteria were determined beforehand [54], with the inclusion criteria being as follows: the paper had to discuss the (potential) use of VRT for smoking cessation treatment; the target population had to be adults, with no pre-existing psychological or physiological conditions; only tobacco smoking in the form of cigarettes, cigars, or pipe and the vaping of nicotine were included (ie, no smoking of crack, marijuana, or any other substance; smoking a nargile or bong; or vaping of tetrahydrocannabinol S containing liquids). After 3 rounds of selection, 42 papers remained. Furthermore, 8 papers that had been identified through the reference list search and met the inclusion and exclusion criteria were added to this selection, as well as 1 meta-review that was published after the search but recommended by the paper reviewer, resulting in a final set of 51 papers. However, because the 2 studies by Pericot-Valverde et al [42,55] are based on the same data set (Pericot-Valverde, personal communication, January 30, 2021), we counted them as 1 study, reducing the total number to 50. Paper selection and data extraction were completed by one of the authors. Figure 1 shows a flowchart overview of the review rounds (see Multimedia Appendix 1 [20,21,42-44,46, 48-52,55-95] for a list of the selected papers).

The timestamped literature selection plan, including search terms and inclusion and exclusion criteria, can be found [54], and the search terms, as well as the raw results from the individual databases, can be found [96] as well as in the Multimedia Appendix 1.

## Results

### Preliminary Analysis

**Figure 2:**
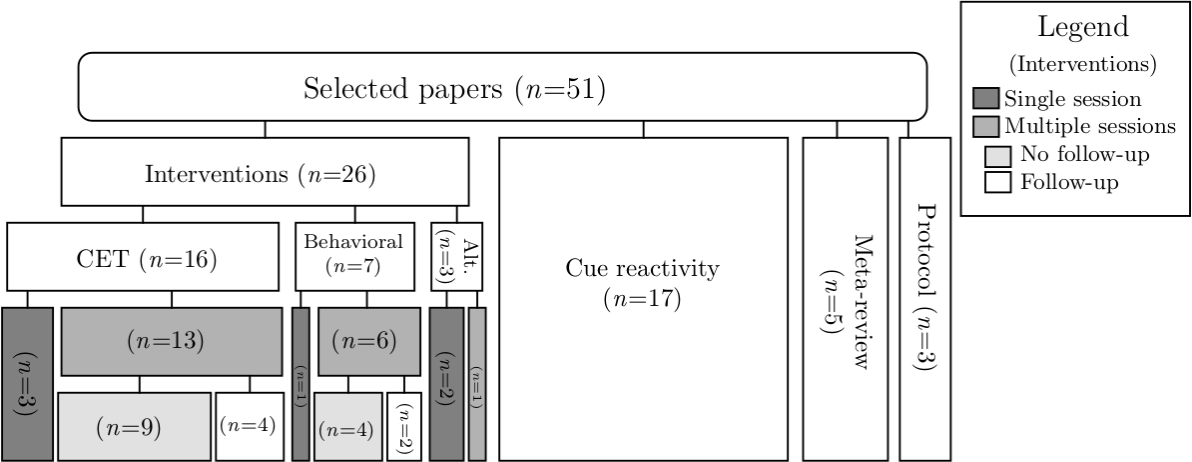
Overview of included studies. Alt: alternative (interventions that are neither based on cue exposure therapy nor behavioral therapy); CET: cue exposure therapy.

A few general observations were made using the data set of papers. Of the 51 selected papers, 26 (51%) introduced or tested an intervention, 17 (33%) studied cue reactivity, 5 (10%) were meta-reviews, and 3 (6%) were protocols for studies yet to be conducted. In addition, 77% (22/26) of the intervention papers described a multisession intervention, stretching out from 3 to 10 weekly sessions. Furthermore, 27% (6/22) of these multisession interventions reported at least 1 follow-up measure, ranging from 1 week to 12 months after the final session. Follow-up measurements are a great asset to intervention studies as initial effects might fade over time (for example, the results in the study by Pericot-Valverde et al [42]). Figure 2 shows a flowchart indicating the division of the papers.

In addition, 54% (14/26) of the interventions were compared with an alternative therapy; however, only 15% (4/26) included a placebo condition. Although not including an alternative intervention or placebo condition does not invalidate the overall findings of a study, it also does not provide any information on the efficacy of one therapy over the other or indeed the efficacy of one therapy over an optimistic mindset.

Furthermore, of the 42 papers examining the effect of an intervention on smoking craving or behavior, 7 (17%) used smokers unmotivated to quit as participants; the rest of the papers was roughly equally divided between smokers motivated to quit (17/42, 40%) and smokers with undisclosed motivation to stop (18/42, 43%). All but 3 papers [44,56,57] used VR in their interventions.

### Has VRT Been Used Satisfactorily to Elicit Smoking Cravings?

The prerequisite for VRT to have any (added) value in smoking interventions is whether or not these techniques can reliably induce craving through activation of smoking and smoking-related associations. Once these cravings have been elicited, any subsequent therapy can target the cravings. Cravings in non-VRT settings are usually elicited by using smoking-related paraphernalia [97], 2D images of cigarettes and scenes with people smoking [19], and imaginary procedures where participants are instructed to imagine themselves in a (personalized) high-risk situation [98]. VR can create a more immersive experience [44-46], which may add to the effectiveness of the therapy, and being virtual, it theoretically allows for tailoring specifically to the user’s needs [42,58].

A plethora of studies (n=17) set out to test whether VR could induce nicotine craving and confirmed that craving can be successfully induced in VR by exposing participants to smoking-related cues [46,58-72] (see the study by Pericot-Valverde [49] for a meta-analysis). Moreover, exposing nonsmokers to VR smoking cues did not elicit cravings [46].

Furthermore, craving can be elicited using minimal cues. No explicit mention of smoking is needed; simply presenting an environment where cigarettes are usually handled (eg, a bar or the checkout counter of a newspaper kiosk) or providing smoking-related cues in the background (such as ashtrays) was sufficient to elicit cravings. The addition of olfactory cues [60,73] or branded cigarette packages [66,67] did not increase the craving response. Ferrer-García et al [74] tested different potential moderators of cue-induced craving and found that the (sense of) presence in VR was positively linked to craving.

Whether VR-cue exposure (CE) elicits craving to a greater extent than the more traditional methods has been less extensively studied. Bordnick et al [70] found that a VR environment elicited greater craving in participants than exposure to a 2D image of the same environment. In contrast, Karelitz [52] conducted a meta-analysis on different factors that may influence cue reactivity and found that although VR cues increased craving, they did not do so to a greater degree than 2D pictorial images or scripted imagery.

It is noteworthy that the number of years that participants had been smoking correlated negatively with cue reactivity [52]. The majority (11/17, 65%) of the cue reactivity studies did not report measuring the number of years smoking; of the 6 that did, 5 did not use it as a control variable or to assess whether the experimental groups were similar before commencing the experiment, thus potentially allowing for bias. Related to this, only 6 of the papers on cue reactivity reported participants’ intention to quit. However, CE supposedly elicits craving in both types of cigarette smokers to an equal extent [62], so this lacuna in the data may not be problematic.

### What VRT Interventions Exist and How Do They Compare to Regular Interventions in Terms of Smoking Outcomes?

#### Overview

Different VRT adaptations of smoking interventions were found. CET in VR (VR-CET) was the most frequently reported (n=16). In addition, VR has been combined with different behavioral interventions (eg, the AAT and gamified behavior training; n=7) and antismoking campaigns (n=3). Figure 2 shows a flowchart detailing the breakdown of the topics.

#### VR-CET Intervention

A total of 16 papers on VR-CET or a variation were included in the review. VR-CET has been successful in reducing smoking cravings over repeated exposures in most experiments [42,58,60,64,68,70,75,76] experiments. One study in the selected papers did not find a significant effect of VR-CET on long-term cravings [77], but their sample size was rather limited at n=8. The studies by Hone-Blanchet et al [50] and Segawa et al [51] present a meta-review on the effectiveness of VR-CET on craving and smoking behavior. A lower amount of smoking behavior after VR-CET treatment compared with the preintervention baseline was reported in the studies by Choi et al [67] and Pericot-Valverde et al [75]. Choi et al [67] measured a reduction from approximately 14 to 4 cigarettes per day based on self-reports, which was substantiated by a significant reduction in exhaled carbon monoxide (CO; 12.5 to 2.5 ppm). Pericot-Valverde et al [75] reported a reduction from 18 cigarettes per day before the intervention to a little under 4 cigarettes per day after the intervention (CO change from 13.2 to 5.0 ppm).

Furthermore, 4 studies investigated the added value of VR-CET to CBT [42,58,61,78]. Culbertson et al [58] compared craving and smoking behaviors between a group of smokers who received CBT and VR-CET and a group of smokers who received CBT and a placebo-VR training. Participants in the VR-CET group not only reported less craving and smoked less after the treatment but also had a significantly higher dropout rate. Thus, it is possible that the results could be biased, as only the participants for whom the VR-CET treatment worked well stayed in the program. Moreover, the intervention effects were only measured directly after intervention.

In addition, 3 studies [42,61,78] did not add a placebo-VR condition but instead compared the craving and smoking behaviors of participants receiving CBT. In the study by Papini et al [78], a group of smokers receiving VR-CET was compared with a group receiving CBT; in the other studies [42,61], both groups received CBT, but only 1 additionally received VR-CET [42,61]. No added value of VR-CET to CBT was found in either of the 3 studies. Furthermore, similar data were found on reduction in dependence and craving [61], abstinence rates at the end of the treatment period [42,78], and decrease in the mean number of cigarettes smoked [78]. In order to further substantiate these findings, Pericot-Valverde et al [42] and Park et al [78] used follow-up measures that confirmed no added benefit of VR-CET to CBT. Relapse at 2 months postintervention was similar between the groups (47% for CBT and 53% for VR-CET) [78]. Pericot-Valverde et al [42] found a significantly higher relapse rate at 12 months postintervention for VR-CET compared with CBT alone (64.3% vs 37%). Relapse rates between 60% and 90% have been reported in the general literature for the first year of quitting. Thus, VR-CET may only marginally improve the odds of smokers successfully giving up their habits.

Three papers described a combination or adaptation of VR-CET with other interventions. First, Kotlyar et al [20] combined VR-CET with NRT by having a third of the participants ingesting a nicotine lozenge before the VR-CET session, thereby artificially reducing the body’s craving for nicotine; the other two-thirds received either a placebo lozenge or none at all. They found that VR-CET combined with both lozenges that contained nicotine and the placebo lozenges reduced self-reported craving compared with no lozenge.

Second, in a similar design, De La Garza et al [21] combined VR-CET with ENDS, where the e-liquid contained different doses of nicotine (0 mg, 8 mg, and 16 mg). Compared with smoking a conventional cigarette, ENDS did not reduce elicited cravings as much, even if they contained a high dose of nicotine. However, the authors noted that the ENDS they used have been known to deliver inferior amounts of nicotine, even with high-nicotine liquids. Moreover, the study did not report whether it controlled for previous ENDS use by the participants.

These results show an interesting pattern in combination with the findings from Moon and Lee [77], who found that even as brain areas associated with addiction and craving became less active over time during VR-CET, participants still reported feeling similar levels of craving. Overall, these studies further underline that bodily craving and subjective feeling of craving only partially overlap, and that nicotine addiction has a strong psychological component as well that a therapeutic intervention should seek to address. This intricate set of motivations for smoking may partially explain why VR-CET as a smoking cessation therapy has resulted in such mixed results. However, more research on the effects of VR-CET in combination with NRT is needed to confirm and nuance this hypothesis. It will be interesting to see the results from one of the protocol papers, which outlined a planned clinical trial that combined VR-CET with NRT (eg, see the study by Papini et al [79]). This study will have a larger number of participants (n=102 vs n=41 [20] and n=7 [21]), and although it does not propose a multisession VR-CET intervention or a follow-up measure, it will still be interesting to see if the results from this intervention are in line with previous findings.

In the third study [80], VR-CET was combined with mindfulness training. Participants were taught mindfulness as a coping mechanism to regulate cravings after CE. Thus, the CET aspect in this intervention moved away from pure extinction in favor of a more cognitive response training component; however, as the authors presented their work as an adaptation of VR-CET, it is listed here as such [80]. This adaptation of VR-CET obtained good results: significantly more (23%) of the mindfulness VR-CET trained participants quit smoking at the end of the intervention compared with the control group (5%), who had received a smoking cessation manual for self-assisted quitting. Furthermore, 3 months after intervention, this significant difference remained, as the percentage of smokers who quit had grown to 33% for the intervention group and remained the same for the control group.

With regard to the entire group of papers on VR-CET, a little over half (n=9) of the papers did not report on participants’ years of smoking. Of the 7 that did, 1 (14%) used it as a covariate and 3 (43%) used it to ensure that the experimental groups were similar. Given how smoking history may have moderated the cue response [74], the 12 papers that did not control for it may have been at risk of bias. About two-thirds of the VR-CET studies (n=11) reported using participants who were motivated to stop smoking; only 1 study targeted smokers who were unmotivated to quit. Although this may not have been problematic for eliciting craving [62], it is unknown whether the motivation to quit affects response extinguishing.

#### Behavioral Interventions

A total of 7 VRT-based smoking interventions that relied on a behavioral component were identified: 1 VR adaptation of the AAT (VR-AAT), 4 papers on gamified behavioral training, and 2 on skill training.

The AAT can be used to measure and break and reverse subconscious biases [23,24]. In a smoking intervention context, this is done by instructing smokers to carry out an avoidance behavior (eg, pushing or swatting away) when confronted with smoking cues, thus reconditioning smoking cues to be associated with avoidance rather than approaching behavior [24]. VR-AAT is a relative newcomer in the list of VR smoking interventions. The 2 papers (supposedly using the same sample, and therefore counted as a single experiment in this review) were published in 2019. As a result, the studies merely functioned as proof of concept for a VR adaptation of the AAT. Indeed, VR-AAT was found to be equally useful for measuring cognitive bias in smokers as regular AAT, and smokers displayed a stronger positive bias toward smoking cues than nonsmokers [81,82].

The 4 gamified interventions all appear to be inspired by the AAT: participants kicked and slapped away cigarette-related cues [56] and crushed cigarettes [83,84] or an alternative *cue*/*reaction* element was incorporated [85].

In a study [56], participants completed 3 weekly sessions in VR and reported on their smoking behavior a week after intervention. No statistical testing of the data was reported; hence, it is not clear if the results were statistically significant.

Participants in the studies by Girard et al [83,84] completed 4 weekly sessions in which they either crushed virtual cigarettes or played a VR placebo game, in addition to smoking cessation counseling. The game significantly reduced smoking behavior as measured directly after the intervention, both in terms of the number of participants who quit and the number of cigarettes smoked by the still-smoking participants [84]. Moreover, 6 months after the intervention, participants who had played the VR crushing cigarette game reported significantly lower craving, as well as smoking fewer cigarettes, whereas a greater number had quit altogether [83]. In addition, fewer participants dropped out from the gamified intervention, and the participants who dropped out did so at a later stage in the program compared with the placebo game [83]. Given that dropout rates in smoking interventions can reach as high as 70% [99] and dropouts are more likely to have higher nicotine dependence and be heavy smokers, especially for early dropouts [99,100], lower dropout rates are a major advantage.

The third gamified VR intervention consisted of 9 weekly sessions [85]. The game is an adaptation of the game proposed by Girard et al [83,84] and has been explicitly designed around the psychological factors underlying motivation. It incorporates elements from both self-determination theory and cue or reaction therapy, thus combining operant (behavior) conditioning with psychological coaching. Although the only data available is a pilot sample (n=8) and no statistical tests have been performed because of the small sample size, the initial results are promising, with lower self-reported dependence as well as a reduction in smoking behavior.

Although these interventions were not phrased as an AAT type of intervention, their results suggest that avoidance behavior training (whether it is pushing away or crushing) could be a promising addition to smoking cessation therapy. What is particularly interesting is the multicomponent approach of the last intervention, which makes it similar to CBT. The behavioral component in CBT, however, is more specific: participants get taught a different response to situations in which they usually give in and smoke, instead of training an automatic, subconscious avoidance response. Although the gamified studies [83,84] included a control group, an aspect that remains unclear is whether VR, the gamification elements, or an interaction between the two were the efficacious components of the interventions.

The 2 VR skill training programs appear to be based more on CBT than on AAT. Bordnick et al [86] combined a 10-week VR coping skill training with NRT and compared this to a standalone NRT intervention. During the VR training sessions, participants were immersed in craving-inducing situations in VR, while the therapist assisted them in identifying high-risk triggers and training coping responses. The addition of VR training reduced smoking rates to a greater degree than NRT alone, both when measured directly after treatment and at the 6-month follow-up. However, similar to the gamified interventions, whether VR was necessary (ie, if it was an improvement over regular CBT) was not tested.

Pericot-Valverde et al [87] described a single case study that used a different CBT-inspired VR intervention, Virtual Stop Smoking therapy. This multicomponent intervention involves self-monitoring, information sessions about smoking, stimulus control procedures, strategies for relapse prevention, problem-solving procedures, strategies to cope with withdrawal syndrome, physiological CO exhalation feedback, and VR exposure, spread out over 6 weekly sessions. The participant had successfully quit smoking by the end of the intervention, but a replication with a larger sample as well as control conditions will be needed before any recommendations can be made.

Of the 7 behavioral interventions described, 2 [83,86] used mean years of smoking as a randomization control variable; none of the other studies reported on the statistic. It is unclear if this factor has an influence on behavioral interventions as it does on cue reactivity. Furthermore, none of the papers reported on using a sample of smokers unmotivated to quit; about half (n=4) used participants motivated to stop smoking.

#### Antismoking Campaigns

Finally, there is the use of VRT in information-based antismoking campaigns that warn about the consequences of smoking. VRT can create a deeper impression than 2D images or movies [88], elicit a stronger negative response, and enhance behavioral intentions to not smoke [44]. However, whether this impact translated to an actual change in behavior was not tested.

### Future Directions

Given the underwhelming results that were achieved by VR-CET in terms of smoking cessation, future studies might want to explore the potential of alternative interventions. However, at present, the interest in VR-CET does not appear to have waned yet, as shown by the number of recent studies that attempt to obtain results by combining VR-CET with other therapies, such as CBT [42,89] and NRT [20,79]. It is possible that combining VR-CET with other interventions will result in a more successful intervention, as multiple aspects of nicotine addiction are addressed.

In contrast to VR-CET, interventions that used some form of behavioral intervention showed promising results. More work will be needed to solidify these findings, especially as a number of the studies reported were merely intended as pilot studies or proof of concept. One protocol study [43] laid out the design for a VR-AAT study, which would be timely as thus far only proof of concept has been reported for VR-AAT interventions. The results from the gamified interventions are promising and could lead to a lower attrition rate.

Possibly because of the ongoing controversy [38,40] surrounding ENDS, few studies to date have explored the potential use of ENDS in combination with VRT for smoking cessation therapy. However, ENDS and VRT both aim to mimic real life and allow for control over the surroundings as well as the amount of nicotine ingested, which could make them an alternative for therapy settings. De La Garza et al [21] reported a pilot study on the combination of VR-CET and ENDS as a form of NRT. The participants indicated that ENDS were not as *satisfying* as the conventional cigarette, even if it contained equal amounts of nicotine; at the same time, the placebo nicotine delivered in the study by Kotlyar et al [20] reduced craving in the same way as the actual nicotine treatment. These findings suggest that craving is a subjective experience and can be tweaked by participants’ beliefs of what relief they will feel. Capitalizing on this make-believe could be an interesting avenue for ENDS-based NRT.

## Discussion

### Principal Findings

The use of VRT could offer an alternative for, or addition to, smoking cessation interventions. VR can be used to recreate triggering situations in a more life-like and persuasive way than traditional methods. This may allow for the creation of an experience where both the environment and the triggers presented are tailored to the user’s circumstances, which may further enhance the effectiveness of the intervention.

Although VR-CET has been the most extensively researched of all interventions, the results have been mixed at best. VR CE reliably elicited cravings, and most VR-CET interventions found that by the end of the therapy, craving in response to smoking-related cues was reduced. However, the effectiveness of therapy above and beyond alternative interventions such as CBT is debatable. These findings echo the conclusions from earlier meta-reviews [48,50,51] and analysis [49]. This lack of results is disappointing but may not be unpredictable as regular CET has been shown to have very limited behavioral effectiveness in battling addictions [22,101]. In fact, this lack of results in conventional CET has been one of the larger motivations for the adoption of VR; extinction of cue reactivity has been shown to be highly context dependent [102], and it has been suggested [46,61] that the discrepancy between the controlled laboratory environment where people were conditioned and the real-world situations where their therapy was put to the test was too large. Introducing VR would supposedly reduce these problems with generalization but still does not yield a significant effect. This may be due to the quality of the virtual environments, as they are far from photorealistic. Addiction remains a multidimensional problem, and CET (in VR or a more traditional setting) may target a component that is too small to be effective as a standalone intervention. Alternatively, the lack of results may be a consequence of the quality of the virtual worlds. Since its introduction, VR has improved greatly, but even as graphics and technology have improved the environments (or at least the ones usually developed for academic studies, ie, on a budget) are far from photorealistic and rarely closely mimic the actual world of the users. Future studies with current VRT may be able to create more realistic and personalized virtual scenarios and thus potentially obtain more reliable results.

Three other types of intervention with a behavioral component emerged: AAT-based [79,80], gamified behavioral training [56,83-85], and skill training [86,87]. The proof of concept has been provided for VR-AAT [81,82], and the 2 immersive games based on AAT demonstrated some success [56,83,84]. Similarly, tentative initial findings suggest a potential for gamified interventions that combine behavioral therapy with psychological coaching [85]. Overall, the potential of gamified interventions as an (effective) treatment program is further enhanced through the addition of a gaming element. Well-designed games lead the user to play out of intrinsic rather than extrinsic motivation [103]; thus, designing an intervention in this format could elicit higher participant engagement, which would, in turn, lead to better therapy compliance and lower dropout rates than conventional interventions. Given the high dropout rates of smoking cessation therapies [104,105] and the importance of compliance in the effectiveness of an intervention [105], these would be major advantages over conventional (nongamified) therapies and interventions.

Only 1 paper was found that combined VR and ENDS in a smoking intervention, possibly because of the ongoing controversy surrounding ENDS [36,37]. Although no consensus has been reached yet on whether the downsides of ENDS outweigh the improvements over conventional cigarettes [38,39], implementing ENDS in future interventions as an NRT component may be explored more as a viable method for smoking cessation.

A final factor that needs consideration in the discussion of VR-based interventions is cost. With the gaming industry’s growing interest in the technology [51], VR has become increasingly accessible over the last few years, with a variety of affordable systems and VR template environments readily available. Nonetheless, developing VRT materials is far from cheap, and the technology thus ought to have a considerable benefit over conventional methods if it is to replace them. Considering this drawback, AR may prove to be an easier and more economical alternative, as it does not require an entire world to be created from scratch but rather uses the existing one as a template to be enhanced or altered.

### Limitations

#### Overview

A few limitations must be noted when interpreting the outcomes of this literature review. Some of these are the consequence of the methodology and design of the papers and interventions reviewed, and some are the result of the methodology of this review itself. All of these are presented in the following section.

#### Limitations of the Reviewed Literature

The first limitation is related to the sample size and sample population reported in the studies. Sample sizes varied widely (between 8 and 102 or 541 for the meta-analysis). Large samples reduce the chance of false negatives and increase the chance of finding small effects [106]. In addition to the size of the sample, not all studies differentiated between treatment-seeking and nontreatment-seeking smokers, which may have been a relevant background variable. Bordnick et al [62] indicated that this may not be a problem for CE–induced craving, but there may still be an effect of motivation to quit on other outcome measures.

The second limitation concerns the experimental design of the studies. The lack of control for potentially confounding variables such as motivation to quit or the number of years of smoking addiction has been mentioned before. Many studies used a 1D measure of effectiveness; for example, some of the VR-CET studies only measured self-reported cravings. Assuming that a therapy or an intervention is considered effective when it has led to a reduction or even cessation of smoking behavior, extrapolating a single-dimension measure to indicate the overall effectiveness of a treatment introduces method bias [107]. Ideally, studies should target more than one type of measure. For example, adding physiological measures for stress (heart rate and skin conductance), smoking behavior (CO exhaled), behavioral measures such as the amount of time the participant is willing to wait until the next cigarette, how much they would be willing to spend on a cigarette, and actual smoking behavior could be included.

Moreover, few studies included control groups, a blinded design, or a follow-up measure. Together with multidimensional measurements that target psychological as well as behavioral responses, these are all methodological aspects fundamental to fully assessing the potential of an intervention. However, these were not implemented in the majority of the studies reviewed in this paper. The lack of assessment of these aspects means that the results obtained in these studies should be interpreted and generalized with care.

#### Limitations of This Review

With regard to this literature review, publication bias [108,109] most likely prevented some studies on VRT smoking interventions from being included in this review. By searching the Cochrane database, at least the study protocols could be found as an indication of the experiments that never made it to publication. It is encouraging that of the 3 protocols that were found with no publication of the results, only one [89] had set out to finish their study before 2020 and could thus conceivably have been published in time for this literature review. Although there is no perfect measure, this can be taken as an indication that publication bias may have been low.

In addition, the literature selection and data extraction were performed by a single researcher, without a second independent researcher confirming the results. Having these decisions depending on 1 person may introduce bias.

### Conclusions

The studies presented in this review suggest that VRT can be considered a promising addition to smoking cessation therapies. Although VR-CET by itself has not yielded consistent results, tentative initial findings on behavioral interventions as well as the combination of VR-CET with these interventions are promising. Moreover, the potential of ENDS in combination with VRT may offer an alternative for future research. More rigorous testing, especially in terms of larger sample sizes, the inclusion of control groups or placebo interventions, and follow-up measures, is still needed.

## Appendix

Multimedia Appendix 1 Overview table with details of all publications selected for this review, including details about the population, intervention method, design, and outcome of the study.

## Abbreviations

AAT: approach-avoidance task
AR: augmented reality
CBT: cognitive behavioral therapy
CE: cue exposure
CET: cue exposure therapy
CO: carbon monoxide
ENDS: Electronic Nicotine Delivery Systems
NRT: nicotine replacement therapy
PRISMA: Preferred Reporting Items for Systematic Reviews and Meta-Analyses
VR: virtual reality
VR-CET: cue exposure therapy in virtual reality
VRT: virtual reality technology
