# Supplementary material for: Virtual Reality Technology Use in Cigarette Craving and Smoking Interventions (I “Virtually” Quit): Systematic Review
Source: J Med Internet Res. 2021 Sep 17;23(9):e24307. doi: 10.2196/24307 (PMC8486991; doi:10.2196/24307)
Supplement: Multimedia Appendix 1 [file jmir_v23i9e24307_app1.docx]

| *Study* | *Population (N)* | *Method* | *Design* | *Outcome* |
| --- | --- | --- | --- | --- |
| [20] | Smokers (*40*) | VR-CET | Randomised within-participant (VR-CET + nicotine administration vs. VR-CET + placebo vs. VR-CET + nothing) single-blind experimental design | Nicotine craving and withdrawal were lower when nicotine or placebo was administered |
| [21] | Tobacco dependent (*7*) | VR-CET | Within participants randomised (conventional cigarette vs. 0mg nicotine vs. 8mg nicotine vs. 16mg nicotine ENDS) design | Smoking conventional cigarette reduced craving better than any ENDS dose |
| [42] | Nicotine dependent (DSM) interested in quitting (*102*) | VR-CET | Randomised controlled trial (VR-CET + CBT vs. CBT) | CET reduced craving, but did not reduced long-term smoking incidence and had higher relapse rates |
| [43] | *n.a.* | VR-AAT | Randomised single-blind between-participant (VR-AAT vs. VR-placebo) design | Protocol; no data reported |
| [44] | Students (*57*) | 3D+AR anti-smoking campaign | Randomised between group (3D+AR images vs 2D images) design | 3D+AR enhanced spatial presence; enhanced negative emotion; and enhanced behavioural intention to spread an anti-smoking message |
| [46] | Smokers (*46*) and non-smokers (*44*) | VR-CE | Mixed randomised (smoker vs. non-smoker; smoking cue vs. neutral cue) design | VR-CE increased craving only with smoking cues and only in smokers |
| [48] | Nicotine dependent (DSM); studies [69, 77, 80, 90] | VR-CET | Meta-review | VR-CET decreases craving over time. Mixed outcomes are reported on whether VR CET adds to NRT in terms of smoking behaviour |
| [49] | Studies [46, 57, 60, 61, 67-69, 71, 72, 74, 83, 91, 104-106](*541*) | VR-CE | Meta-analysis | VR-CE reliably elicits cravings, even when publication bias is taken into account |
| [50] | Studies [46, 65, 67-69, 71-75, 79, 80, 91, 105, 106, 108] | VR-CET | Meta-review | VR-CE reliably induces craving, but contradictory findings on whether VR-CET reduces craving |
| [51] | Studies [42, 46, 61, 66-68, 70, 73-75, 77, 80, 85, 90, 103-105] | VR-CET | Meta-review | Over the short term, VR-CET consistently increases craving. Mixed effectiveness for decreasing craving in the long term and smoking behaviour |
| [52] | 53 studies on cue reactivity (also without VR) [61, 68, 75] (*3372*) | VR-CE | Meta-regression on cue reactivity, with 'mean years smoking', 'cue mode' (images, scripted imagery, video, VR, in vivo), 'abstinence' (yes/no) as predictors | Mean years of smoking was negatively related to reactivity effect size, cue mode was related to reactivity effect size with images and scripted imagery being related to larger effect sizes than video, in vivo, and VR. |
| [56] | Smokers who recently quit (*61*) | Partially immersive game | Quasi-randomised between participants (game intervention vs. waiting list) | No significance testing on the findings |
| [57] | Female smokers (*32*) | 3D immersive environment | Within participant design (pleasant cues vs. unpleasant cues vs. smoking related cues) | Smoking related VR-CE elicited similar psycho-physiological responses as unpleasant VR-CE, but the smoking related cues were subjectively evaluated as positive |
| [60] | Nicotine dependent (DSM) treatment seeking (*15*) | VR-CET | Randomised between participant single blind (CBT + VR-CET vs. CBT + VR-placebo) | CBT + VR-CET had higher quit rates and lower smoking incidence than CBT + VR-placebo at end of treatment |
| [61] | Nicotine dependent, not motivated to quit (*36*) | VR-CE | Within participant randomised (neutral cues vs. smoking related visual + olfactory cues) | Smoking related VR-CE elicited more craving |
| [62] | Nicotine dependent (DSM) young adult (*20*) | VR-CE | Within participant (social cues vs. object cue) design | Both smoking cues increased craving compared to baseline. No added effect of olfactory cues |
| [63] | Nicotine dependent (DSM) abstinent for 7+ days (*61*) | VR-CET | Randomised between-participant (VR-CET + CBT vs. CBT) | VR-CET did not reduce smoking behaviour |
| [64] | Nicotine dependent (DSM) treatment seeking and not motivated to quit (*104*) | VR-CE | Between participant (treatment seeking vs. non-treatment seeking) design | Craving was induced in both treatment-seeking and non-treatment seeking smokers |
| [65] | Nicotine dependent not motivated to quit (*47*) | VR-CE | Pre-post VR cue exposure testing | Post VR-CE cravings were stronger than pre VR-CE cravings |
| [66] | Nicotine dependent (DSM) treatment seeking (*46*) | VR-NRT | Randomised within participant (smoking cues vs. neutral cues) repeated measure (10 weekly sessions) design | Smoking cues elicited greater craving than neutral cues. Over the intervention period the craving response reduced |
| [67] | Regular smokers, not motivated to quit (*46*) | VR-CE | Pre-post intervention testing | Smoking cues induced craving |
| [68] | Daily smokers (*24*) | VR-CE | Within participant (explicit smoking cues vs. implicit smoking cues vs. neutral cues) | Compared to the baseline, both VR environments increased craving |
| [69] | Nicotine dependent treatment seeking (*10*) | VR-CET | Within participant repeated (4 weekly sessions) measure (social smoking cues vs. object smoking cues) design | Both social and object smoking cues induced craving. Over the sessions craving decreased, as well as smoking behaviour |
| [70] | Former smokers (*25*) | VR-CE | Within participant (seven VR rooms with smoking cues) design | All VR-CE environments induced craving |
| [71] | Nicotine dependent, not motivated to quit (*10*) | VR-CE | Controlled experimental trial, within-participant (smoking cue with vs. without social interaction) design | Social interaction in a VR-CE session did not enhance craving |
| [72] | Nicotine dependent, not motivated to quit (*13*) | VR-CET | Within participant (smoking cue v. neutral cue) in randomised order | VR smoking cues increased craving compared to neutral cues |
| [73] | Male smokers (*22*) | VR-CET | Randomised between participant (VR vs. 2D cues) design | VR cues increased craving compared to baseline; 2D cues did not |
| [74] | Smokers (*45*) | VR-CE | Randomised within-participant (mimic smoking in VR, mimic darts in VR, no activity in VR) design | VR-CE with smoking cues increased craving compared to other two conditions |
| [75] | Nicotine dependent (DSM) young adults (*20*) | VR-CE | Randomised mixed 2 (within participant: neutral cues vs. smoking cues) by 2 (between participants: olfactory cues vs. no olfactory cues) design | Smoking cues increased thoughts about smoking and attention to cigarettes. No effect from olfactory cues |
| [76] | Smokers (*46*) | VR-CE | Correlation study on moderators (nicotine dependence, anxiety, impulsiveness, presence in VR) | Only presence related to higher post VR-CE craving |
| [77] | Nicotine dependent (DSM) treatment seeking (*48*) | VR-CET | Pre-post intervention (5 weekly sessions) testing | Post-intervention smoking behaviour and craving was lower than at pre-intervention |
| [78] | Nicotine dependent (DSM) motivated to quit (*32*) | VR-CET | Pre-post intervention (5 weekly VR-CET sessions) testing | Craving was lower post-treatment than pre-treatment |
| [79] | Adolescent smokers (*8*) | VR-CET | Repeated measure (six sessions) | No reduction in subjective craving over time; but smoking behaviour decreased slightly. Brain areas associated with craving became less active post-intervention |
| [80] | Male treatment seeking smokers (*30*) | VR-CET | Randomised between participant 2 (pre- vs. post-intervention) by 2 (VR-CET vs. CBT) design | Smoking behaviour and subjective dependence were lower post-treatment than pre-treatment and at 2-month follow up for both interventions; no difference for intervention type |
| [81] | *n.a.* | VR-CET | Randomised double-blind between-participant (isradipine + VR-CET vs. placebo + VR-CET) | Protocol; no data reported |
| [82] | Smokers interested to quit (*120*) | VR-CET | Randomised controlled between participant design (VR-CET with mindfulness training vs. standard intervention manual | Mindful VR-CET had higher self-reported abstinence at end of the program as well as 3-month follow up than the group receiving standard care |
| [83]; also [84] | Nonsmokers (*15*) and smokers (*5*) | VR-AAT | Mixed design: between (smoker vs non-smoker) and within (VR-AAT vs AAT) factor | Smokers exhibit a stronger cognitive bias to smoking-related cues than non-smokers |
| [85] | Regular smokers, treatment seeking (*91*) | VR behaviour game | Randomised between participant (counselling + intervention game vs. counselling + placebo game) design | The intervention game decreased subjective nicotine addiction, decreased smoking incidence, and increased abstinence rates post-treatment and 6 months follow-up. Drop-out rates were lower |
| [86] | Heavy smokers, treatment seeking (*16*) | VR behaviour game | Pre-post intervention comparison of an AAT-like game | Number of cigarettes smoked was lower post-intervention |
| [87] | Cigarette smokers (*8*) | VR video game intervention | Pre-post intervention (9 weekly sessions) testing | Decrease in smoking behaviour and self-reported dependence; no significance testing due to small sample |
| [90] | Nicotine dependent (DSM) treatment seeking (*86*) | VR skill training | Between participant (VR skill training + NRT vs. NRT) | VR skill training + NRT reduced craving and smoking rates compared to NRT |
| [91] | Smoker (*1*) | VSS | Case report | Subject stopped smoking over the duration of 6 weekly sessions |
| [92] | Young adult smokers, unmotivated to quit (*40*) | VR warning intervention | Randomised cross-over trial, within participant, 3 conditions (VR anti-smoking intervention vs. anti-smoking video vs. shocking images on cigarette pack) | Compared to pre-intervention baseline, VR > video > shocking image for increasing the motivation to quit |
| [93] | *n.a.* | VR-CET | Randomised controlled between participants (VR-CET + CBT vs. Progressive Muscle Relaxation and CBT) design | Protocol; no data reported |
| [103] | Nicotine dependent (DSM) treatment seeking (*48*) | VR-CET | Pre-post intervention (5 weekly VR-CET sessions) testing | No significance testing |
| [104] | Young adult smokers (*34*) and non-smokers (*21*) | VR-CE | Between participant (neutral cues vs. smoking cues) randomised | Smoking cues increase craving in smokers but not in nonsmokers |
| [105] | Nicotine dependent (DSM) young adults not motivated to quit (*22*) | VR-CE | Within participant (smoking cues vs. neutral cues) design | Higher craving after smoking cue exposure |
| [106] | Smokers (*20*) | VR-CE | Within participant (smoking cue vs. neutral cue) design | VR-CE with smoking cues created larger craving than VR-CE with neutral cues |
| [107] | Nicotine dependent (DSM) treatment seeking (*102*) **NB** uses same dataset as [42] | VR-CET | Randomised clinical trial; between participant (VR-CET + CBT vs. CBT) design | No difference in abstinence between groups |
| [108] | Student smokers (*32*) and non-smokers (*28*) | VR-CE | Randomised mixed design (smoker vs. non-smoker; explicit smoking cues vs. neutral cues) | VR-CE with explicit smoking cues increased craving in smokers |
| [109] | Student smokers and non-smokers (*13*) | VR-CE | Mixed design (smoker vs. non-smoker; and smoking cues vs. neutral cues) | EEG measures showed smokers had higher frontal activity when confronted with smoking-related cues, compared to neutral cues, and compared to non-smokers |
